# Antiproliferative Activities of Methanolic Extract and Fractions of *Tetrapleura Tetraptera* Fruit

**DOI:** 10.1155/2021/4051555

**Published:** 2021-07-19

**Authors:** Anastasia Rosebud Aikins, Peggy Afua Birikorang, Mary Chama, Eunice Dotse, Abigail Anning, Regina Appiah-Opong

**Affiliations:** ^1^West African Centre for Cell Biology of Infectious Pathogens, Department of Biochemistry, Cell and Molecular Biology, University of Ghana, Accra, Ghana; ^2^Department of Chemistry, School of Physical and Mathematical Sciences, University of Ghana, Accra, Ghana; ^3^Department of Clinical Pathology, Noguchi Memorial Institute for Medical Research, University of Ghana, Accra, Ghana

## Abstract

Most of the current cancer chemotherapeutics are associated with harsh and undesirable side effects, including toxicity and chemoresistance, driving the need for safer and more effective alternatives. In this study, the antiproliferative activities of the methanolic extract of *Tetrapleura tetraptera* fruits and nine different fractions (C1–C9) from the column chromatographic separation of the extract against leukemia (Jurkat) and human breast cancer (MCF-7) cell lines were investigated using a tetrazolium-based colorimetric assay. Phytochemical screening of the extract and fractions found alkaloids, carbohydrates, flavonoids, glycosides, phenols, saponins, steroids, tannins, and terpenoids in the methanolic extract. Most of the fractions exhibited antiproliferative activity (>100 *μ*g/mL) with the Jurkat cells being more susceptible than the MCF-7 cells. Four of the collected fractions C4, C3, C5, and C2 had good selective indices in decreasing order of activity, in the case of Jurkat cells. Liquid chromatography-mass spectrometry analysis of all samples (except for C4 and C9) revealed that C1, C2, C3, and C5 each had a single component. More importantly, fractions C2, C3, and C5, which were selective to Jurkat cells, also had the same retention time of 1.846 min. Fractions C6 and C8 had two components, with C7 having four components. This study serves as a basis for further work to isolate and characterize potential anticancer agents from the fractions of extracts of *T. tetraptera* fruits.

## 1. Introduction

Cancer remains a global health challenge, with about 18.1 million new cases and 9.6 million deaths recorded annually [1]. Most of the available therapies or interventions for cancer treatment are accompanied by side effects such as hair loss, numbness or chronic pain in some parts of the body, damage to vital organs, chemoresistance, and tumor reoccurrence [2, 3]. Cancer research, therefore, continues to be focused on the development of new therapies with little to no side effects and/or improvement of existing therapies. Exploring the use of natural products, especially plant parts, is a major area of focus [4].

*Tetrapleura tetraptera* is a perennial plant that is widespread in tropical Africa, particularly Ghana, where it is found mostly in the northern part of the country and its fruit is locally known as “prekese.” The most common use of the fruit in Ghana is adding it to food as a spice because of its unique aroma. *T. tetraptera* is one of the most medicinally beneficial plants because of its several biological activities. Notable among them are its antioxidant [5, 6], anti-inflammatory [7], antimicrobial [8], hypotensive [9], hypoglycemic, and antidiabetic activities [10].

Studies involving the anticancer effects of *T. tetraptera* have mainly focused on crude extracts. For instance, studies have shown that the methanolic extract of *T. tetraptera* fruit exhibits potent anticancer activity against human breast cancer cell lines, BT-549 and BT-20, and the T-lymphoblastic leukemia cell line, Jurkat [11]. In addition, Kuete et al. (2011) showed that the methanolic extract had anticancer activity against MiaPaCa-2, a human pancreatic cancer cell line, and the leukemia cell lines, CCRF-CEM and CEM/ADR5000 [12]. The ethanolic extract of *T. tetraptera* fruit also exhibited strong antiproliferative activities against Ehrlich ascites carcinoma both in vitro and in vivo [13]. Anticancer studies involving fractionation of the extracts of *T. tetraptera* would help better elucidate the components of the crude extract with antiproliferative activity.

In this study, we investigated the antiproliferative activities of the crude extract and column chromatography-derived fractions of *T. tetraptera* fruit against two human cancer cell lines as a measure of anticancer activity.

## 2. Materials and Methods

### 2.1. Plant Collection and Preparation

*T. tetraptera* fruits were purchased from the Madina market in Accra, Ghana, and authenticated by a taxonomist at the University of Ghana Herbarium, Department of Plant and Environmental Biology. The fruits were washed thoroughly and oven-dried at 65°C for 4 h. The dried fruits were pulverized and stored at 4°C in an airtight container until use.

### 2.2. Extraction and Fractionation of the Crude Methanolic Extract

Five hundred milliliters of 99% methanol was used to extract the compounds in 100 g of the pulverized fruit using the Soxhlet extraction method for 10 h. The resulting extract was filtered and concentrated using a rotary evaporator.

### 2.3. Fractionation of the Extract Using Column Chromatography

A glass column preloaded with a slurry of silica gel and chloroform was loaded with the methanolic extract. Beginning with 100% chloroform, the ratio of chloroform to methanol was varied as the columns were progressively run until 100% methanol was used for the final elution. Volumes of 20 mL eluates were collected in test tubes. Based on the thin layer chromatography of the fractions, they were combined and concentrated to dryness using rotary evaporation under vacuum. The resulting residues were reconstituted in dimethyl sulfoxide (DMSO) and stored at −4°C until use.

### 2.4. Qualitative Phytochemical Analysis

Qualitative phytochemical screening was performed on the crude extract and fractions as described by Trease and Evans [14]. The phytochemicals that were tested were terpenoids, flavonoids, alkaloids, glycosides, carotenoids, tannins, saponins, phenols, steroids, and carbohydrates. The methods used to ascertain the presence or absence of these phytochemicals are briefly described below.

### 2.5. Alkaloids

A few drops of a saturated picric acid solution were added to 2 mL of a solution of the crude extract or fractions. The formation of yellow precipitates indicates the presence of alkaloids.

### 2.6. Carbohydrates

Two milliliters of Fehling's solutions A and B was added to 2 mL of the crude extract or fractions and heated until boiling. The presence of carbohydrates was indicated by the formation of brick-red precipitates.

### 2.7. Carotenoids

A few drops of chloroform followed by sulfuric acid were added to 2 mL of the crude extract or fractions. The blue color formation at the interface showed the presence of carotenoids.

### 2.8. Flavonoids

Three drops of ferric chloride solution were added to 2 mL aliquots of the test samples, and the presence of flavonoids was ascertained by the formation of a blackish-red color.

### 2.9. Glycosides

Concentrated sulfuric acid (2 mL) was added to 2 mL of each test sample. A reddish-brown coloration indicated the presence of glycosides.

### 2.10. Phenols

A few drops of lead acetate were added to 2 mL of the crude extract or fractions, and the formation of a yellow precipitate showed the presence of phenols.

### 2.11. Saponins

Two milliliters of the extract or fractions was mixed with approximately 3 mL of water. Upon shaking, the formation of foam, which was stable for 15 min, showed the presence of saponins.

### 2.12. Steroids

Two milliliters of chloroform was added to 2 mL of each sample, and then 2 mL of chloroform was added, followed by a few drops of concentrated sulfuric acid. The development of a red layer in the test tube confirmed the presence of steroids.

### 2.13. Tannins

A few drops of basic lead acetate solution were added to 2 mL of the crude extract or fractions, and the formation of a white precipitate indicated the presence of tannins.

### 2.14. Terpenoids

Two milliliters of chloroform was added to 2 mL aliquots of the sample, and then 2 mL of chloroform was added. The resultant mixture was evaporated to dryness, and a few drops of sulfuric acid were added and heated for approximately 2 min. The development of a gray color indicates the presence of terpenoids.

### 2.15. Cell Culture

The cell lines used in this study were the human breast cancer cell line, MCF-7, T-lymphoblastic leukemia cell line, Jurkat, and Chang liver cells (derived from HeLa cells but used in place of normal cells due to their slow proliferative properties). Jurkat and Chang liver cell lines were cultured in RPMI-1640 medium, and MCF-7 cells were cultured in DMEM. Both culture media were supplemented with 10% fetal bovine serum (FBS) and 1% penicillin-streptomycin. The cells were maintained in a humidified incubator at 37°C and 5% CO_2_ with periodic changes in media and passaging until they were ready for use. These cells were seeded into 96-well microtiter plates at a density of 1 × 10^4^ cells per well in a 100 *μ*L volume for 24 h prior to treatment.

### 2.16. Treatment of Seeded Cells

Five different concentrations of the crude extract and each of the fractions were prepared from stock solutions using 1% DMSO as the solvent. They were added to the seeded wells at a final concentration of 1000, 500, 250, 125, and 62.5 *μ*g/mL in triplicate. Curcumin was used as a standard compound for the positive control. For the wells designated for use as a positive control, 10 *μ*L of five different concentrations of curcumin was added to obtain final concentrations ranging from 2 to 38 *μ*g/mL. DMEM containing 1% DMSO was used as the negative control. The treated cells were then incubated for 72 h.

### 2.17. Cytotoxicity Assay

The 3-(4, 5-dimethylthiazol-2-yl)-2, 5-diphenyltetrazolium bromide (MTT) assay was performed to measure the effect of the crude extract and fractions on the proliferation of MCF-7 and Jurkat cells. The effect of the extracts was assessed using Chang liver cells to calculate the selectivity index. After 72 h of incubation of treated cells, 20 *μ*L of 2.5 mg/mL MTT was added to each well in a 96-well culture plate and further incubated in the dark for 4 h. The reactions were stopped by adding 150 *μ*L of acidified isopropanol. Subsequently, the cells were incubated in the dark at room temperature (26°C) overnight. Absorbance was read at a wavelength of 570 nm using a fluorescence microplate reader (Tecan Infinite M200, Austria).

From the absorbance values obtained, percentage cell viabilities were calculated for the various concentrations of the fractions, crude extract, and curcumin using the following formula:

(1) $$\begin{array}{c} \text{percentage cell viability}=\frac{\text{absorbance of treated wells}-\text{absorbance of color control}}{\text{absorbance of untreated wells}-\text{absorbance of blank}}. \end{array}$$

Graphs of percentage viability against concentration were plotted for each fraction, crude extract, and curcumin for the three cell lines. Inhibition concentrations at 50% (IC_50_) were obtained from these graphs. The IC_50_ value is the concentration of the test compound required to reduce cell viability by 50%. These IC_50_ values were used to calculate the selectivity indices using the following formula:

(2) $$\begin{array}{c} \text{selectivity index }=\;\frac{{\text{IC}}_{50}\text{ of treated normal cell lines}}{{\text{IC}}_{50}\text{ of treated cancer cells}}. \end{array}$$

The selectivity index (I) is a measure of the cytotoxic selectivity of drugs or extracts, which implies their ability to differentiate and target cancer cells with little to no harm to nonmalignant cells. Drug candidates with SI ≥2 were considered to have good therapeutic abilities.

### 2.18. Liquid Chromatography-Mass Spectrometry (LC-MS) of the Crude Extract and Fractions

Low-resolution ESi-MS data were acquired on an Agilent 1260 Infinity HPLC system (Agilent® 1260 Infinity Binary Pump, Agilent® 1260 Infinity Diode Array Detector (DAD), Agilent® 1290 Infinity Column Compartment, and Agilent® 1260 Infinity Standard Autosampler) coupled to an Agilent 6120 Quadrupole MS system and Peak Scientific® Genius 1050 nitrogen generator. A Phenomenex Kinetex® 2.6 *μ*m EVO C18 100 Å (30 × 2.1 mm) reverse-phase analytical column was used. The chromatographic method included a column temperature of 40°C, an injection volume of 2 *μ*L, a flow rate of 0.7 mL/min, and maximum column backpressure set at 600 bars. The mobile phase consisted of 10 mM NH_4_OAc in water (A) and 10 mM NH_4_OAc in methanol (B). The LC run is a 4.50 min duration beginning with 15% a of B from 0 to 0.30 min, followed by a speedy increase in the gradient to 100% B over 0.90 min. The mobile phase composition at 100% B was maintained at 4.50 min. Mass spectra were collected from *m*/*z* 100–800 in both the negative and positive modes.

### 2.19. Statistical Analysis

The data were analyzed using GraphPad Prism version 8 and the 2013 version of Microsoft Excel. The data are expressed as the mean ± SD.

## 3. Results

### 3.1. Qualitative Phytochemical Screening of Crude Extract and Fractions

All phytochemicals tested, including alkaloids, carbohydrates, flavonoids, flavonoids, glycosides, phenols, saponins, steroids, tannins, and terpenoids, were present in the crude extract, except for carotenoids, which were also absent in the fractions (Table 1). The first fraction, C1, did not indicate the presence of any phytochemicals tested. Among the fractions, steroids were only present in C2, terpenoids were present in C3 and C5, and phenols were present in C3 and C6. Flavonoids and saponins were present in fractions C6–C9.

### 3.2. Cytotoxicity (MTT) Assay

**Figure 1:**
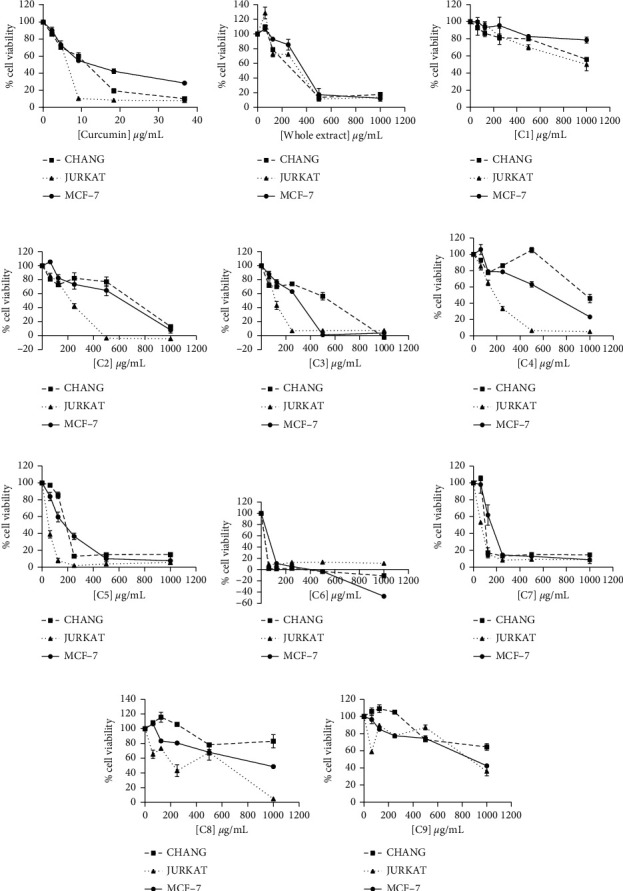
Dose-response curves of percentage viability against the concentration of *T. tetraptera* whole extract and its fractions and curcumin on Chang liver, Jurkat, and MCF-7 cell lines.

The effect of the crude extract and fractions of *T. tetraptera* fruit on the proliferation of the cell lines was determined using the MTT assay. Dose-response curves plotted as percentage viability against the concentration of fractions and crude extract are shown in Figure 1. Fractions C5, C6, and C7 exhibited higher cytotoxicity in the three cell lines, similar to curcumin, whereas fraction C1 did not have much effect on the cell lines even at a concentration of 1000 *μ*g/mL (Figure 1).

### 3.3. Inhibition Concentration at 50% (IC50) Values

The IC_50_ values of the extracts and fractions are presented in Table 2. The crude extract and fractions were more cytotoxic to Jurkat than MCF-7 cells, showing lower IC_50_ values in Jurkat than MCF-7 cells.

Fraction 1 (C1) was inactive because it had IC_50_ values >1000 *μ*g/mL in all cell lines. For both Jurkat and MCF-7 cells, fraction 6 had the strongest cytotoxic activity, with the lowest IC_50_ values of 35.85 ± 2.29 *μ*g/mL and 70.07 ± 0.75 *μ*g/mL, respectively, in Jurkat and MCF-7 cells. In Chang liver cells, fraction 6 was also cytotoxic with an IC_50_ value of 31.86 ± 0.31 *μ*g/mL.

### 3.4. Selectivity Indices

From the IC_50_ values obtained for the crude extract and fractions against the three cell lines, selectivity indices (SI) were calculated as a measure of their ability to differentiate malignant from nonmalignant cells in exerting their antiproliferative effects. Fractions with SI values above the 2.0 threshold were considered to have therapeutic activity, whereas those below were considered toxic.

**Figure 2:**
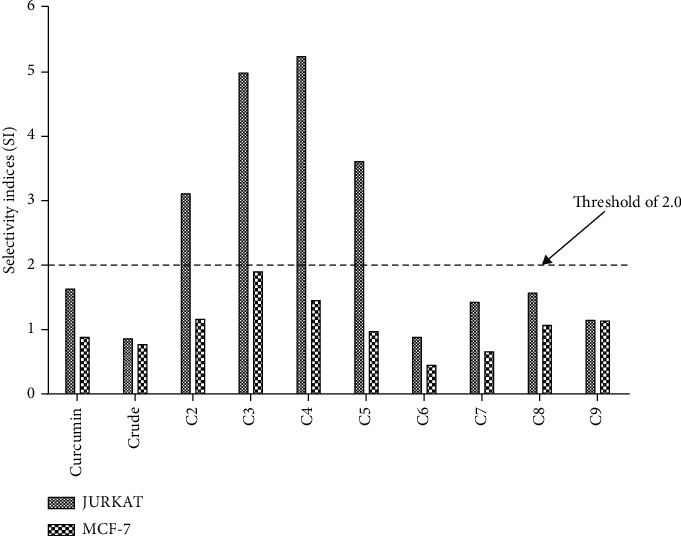
Selectivity indices of crude extract (crude), fractions, and curcumin.

Figure 2 shows that none of the fractions or crude extract had therapeutic activity against MCF-7 cells, as they had SI values less than 2. However, against Jurkat cells, four fractions had SI values greater than 2, indicating therapeutic ability. These fractions are 4, 3, 5, and 2 in decreasing order of activity. The crude extract had SI values less than 2 in both Jurkat and MCF-7 cell lines. Fraction 6, which had the lowest IC_50_ values in both cell lines, had the lowest SI values in both cancer cell lines, indicating poor therapeutic potential.

### 3.5. LC-MS Analysis of Crude Methanolic Extract and Fractions

**Figure 3:**
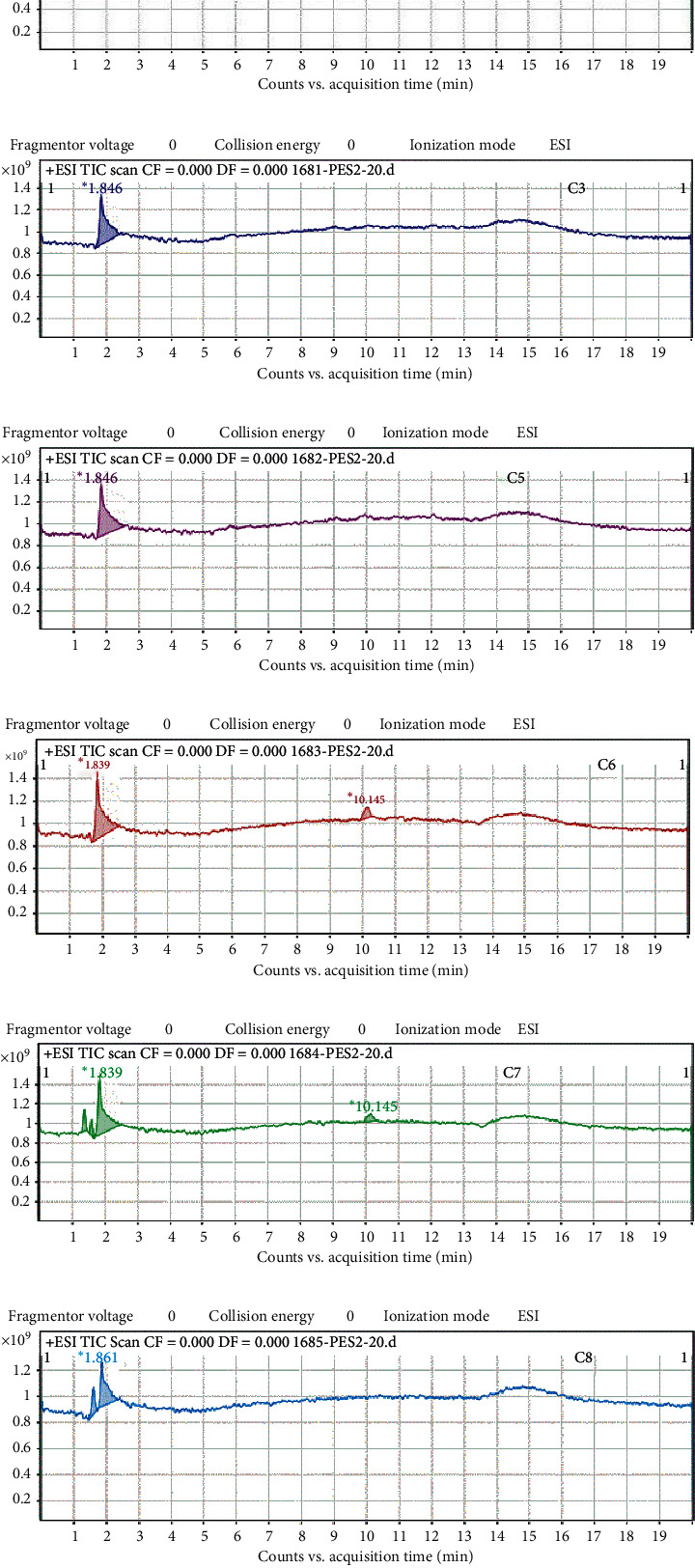
(a) LC-MS chromatogram of fraction C1 of *T*. *tetraptera*. (b) LC-MS chromatogram of fraction C2 of *T*. *tetraptera*. (c) LC-MS chromatogram of fraction C3 of *T*. *tetraptera*. (d) LC-MS chromatogram of fraction C5 of *T*. *tetraptera*. (e) LC-MS chromatogram of fraction C6 of *T*. *tetraptera*. (f) LC-MS chromatogram of fraction C7 of *T*. *tetraptera*. (g) LC-MS chromatogram of fraction C8 of *T*. *tetraptera*. (h) LC-MS chromatogram of the crude methanolic extract of *T*. *tetraptera.*

Fractions C1–C3 and C5–C8 obtained from the chromatographic separation of the crude methanolic extract were analyzed for their various components using liquid chromatography-mass spectrometry (LC-MS). Each of the fractions C1–C3 and C5 had single components with C1 eluted at a retention time of 1.839 min and the rest at 1.846 min (Table 3 and Figure 3). The same retention times and fragment ions were obtained for components C2, C3, and C5 suggesting that these three fractions are the same. Fractions C6 and C8 had two components each, whereas fraction C7 had four components. The component in fraction C6 eluted at 1.839 min (87.3%) was also eluted in C1 (100%) with similar fragmentation, while the one at 10.145 min (12.7%) was present in C7 (9.69%). The crude extract showed a peak at 1.853 min (100%).

## 4. Discussion

One of the major limitations of most cancer therapies used in recent times is the accompanying side effects, including damage to vital organs and tumor reoccurrence. Research into the efficacy of natural compounds, especially products from plants as anticancer agents, continues to be of paramount importance [15]. Over the years, research has shown that some plants have potent anticancer activities. For instance, the crude extract from the *Allium wallichii* plant has anticancer activity against prostate, breast, and cervical cancer cell lines [16]. The extract from the stem bark of *Zanthoxylum alatum* is also cytotoxic against human lung and pancreatic cancer cell lines [17].

Our study sought to investigate the antiproliferative potential of the crude methanolic extract and fractions of the fruit of *T. Tetraptera*, derived from column chromatography, against human MCF-7 breast cancer and Jurkat leukemia cell lines. *T. tetraptera* is known to contain various phytochemicals. Most phytochemicals are produced by plants for protective roles, thereby conferring one or more biological activities to the plant.

Phytochemicals found in the fruit of *T. tetraptera* include alkaloids, saponins, tannins, flavonoids, reducing sugars, glycosides, terpenoids, phenols, steroids, and anthraquinones [6, 18, 19]. Two oleanane-type saponins, tetrapteroside A and tetrapteroside B, have been isolated from the stem bark of plants [20]. The leaves and stems also contain stigmasterol, stigma-5, 22-diene-3-O-*β*-D-glucopyranoside, 3-O-*β*-D-glucopyranosyl-2ʹ-acetamido-2ʹ -deoxy]-oleanolic acid, pheophytin, and tetracosanol [21]. The high saponin content, for instance, confers potent antimicrobial activity because saponins are produced to deter foreign pathogens from attacking and destroying the plant [22]. The flavonoids scopoletin, 2ʹ, 4, 4ʹ-trihydroxychalcone isoliquiritigenin, 2ʹ, 3, 4, 4ʹ-tetrahydroxychalcone-butein, and 4ʹ, 5, 7-trihydroxyflavanone-naringenin have also been isolated from the fruits of *T. tetrapleura* [23, 24]. Furthermore, phytic acids, oxalates, and cyanogenic glycosides are also present in the fruit [25].

In our study, apart from carotenoids that were not found in the crude extract and fractions, the other phytochemicals were tested; alkaloids, carbohydrates, flavonoids, glycosides, phenols, saponins, steroids, tannins, and terpenoids were present, consistent with the findings of other studies [26, 27].

Various phytochemicals, especially polyphenols, including phenolic acids, flavonoids, and stilbenes, have been shown to possess potent anticancer activities [28]. For instance, resveratrol (a stilbenoid) has potent anticancer activity against various cancer types, including human colorectal cancer [29], while curcumin is potent against various cancers, including human liver cancer [30]. Epigallocatechin-3-gallate, one of the most important tannins found in green tea, has also been reported to have potent apoptotic, antiproliferative, and antimetastatic effects in various human cancers, such as liver, lung, and ovarian cancers [31–33]. Similarly, flavonoids, such as quercetin, have been reported to exhibit anticancer effects in prostate cancer [34]. Thus, the phytochemicals in the crude extract and fractions might be responsible for the observed antiproliferative effect on the cancer cells in this study.

The crude extract had antiproliferative activity against both MCF-7 cells and the acute T cell leukemia cell line, Jurkat, exhibiting a more cytotoxic effect on Jurkat than MCF-7 cells. This finding is consistent with the findings of other studies. For instance, the methanolic extract of *T. tetraptera* fruit showed strong cytotoxicity against two human breast cancer cell lines: BT-549 and BT-20 as well as Jurkat with IC_50_ values of 9.1, 23.1, and 37.5 *μ*g/mL, respectively [11]. It has also been shown that extracts from the fruit had cytotoxic effects against CCRF-CEM leukemia cells and MDA-MB-231-pcDNA3 breast cancer cells as well as their drug-resistant variants, CEM/ADR5000 and MDA-MD-231-BCRP, with IC_50_ values of 10.27, 20.47, 17.16, and 15.75 *μ*g/mL, respectively [35]. In another study, the leukemia cell lines, CCRF-CEM and CEM/ADR5000, and the pancreatic cancer cell line, MiaPaCa-2, showed cytotoxicity, although it was not as potent as some of the other plants tested [12]. *T. tetraptera* fruit has lower amounts of tannins, saponins, phenols, and sterols with higher contents of alkaloids, flavonoids, and hydrogen cyanide, with flavonoids being the most abundant [36]. The overall anticancer activity of the samples that showed antiproliferative activity in this study might be due to the abundance of flavonoids.

Compounds with promising therapeutic value must be able to selectively target cancer cells without affecting normal cells [37]. An anticancer agent is considered to have pharmacological or therapeutic activity if it has an SI value greater than or equal to 2 [38, 39]. Compounds or drugs with SI values less than 2 are therefore considered toxic as they harm normal cells as much as or more than cancerous cells. Three out of four fractions with good selectivity indices, C2, C4, and C5, contained glycosides, another member of the polyphenol group. Indeed, some glycosides have been reported to exhibit potent anticancer activities. Schneider et al. reported that a group of glycosides, referred to as cardiac glycosides, have potent anticancer activities against various types of human cancers, including breast cancer [40].

Despite its potent antiproliferative activity, C6 is cytotoxic to cancer cells and hence not suitable for consideration in chemotherapeutic development. Phytochemicals present in the most cytotoxic fraction, C6, are flavonoids, alkaloids, tannins, saponins, phenols, and carbohydrates. However, the fractions that were most selective to the cells (C2, C3, C4, and C5) did not contain flavonoids and saponins. Further studies are needed to determine whether the absence of flavonoids and saponins accounts for the good selectivity indices of these fractions.

From the LC-MS analysis, the same retention time (RT) of 1.846 min was obtained for fractions C2, C3, and C5, suggesting that they may be the same, which explains why they were all selective against Jurkat cells. Further studies to elucidate the active compounds in these fractions might contribute to the development of improved agents for cancer, especially leukemia.

Altogether, our findings underscore the importance of fractionating crude extracts to help isolate compounds that are selective only for cancer cells to minimize toxicity against normal cells.

## 5. Conclusion

In conclusion, our findings are consistent with those from other studies on the prospects of *T. tetraptera* in the quest for natural and less harmful therapies for cancer. More importantly, our findings showed that some fractions from the methanolic extract had better antiproliferative activity than the crude extract. Our findings also underscore the importance of fractionating the crude extract to help isolate compounds that are selective only for cancer cells to minimize toxicity against normal cells.

In the present study, identification of the main components in the fractions with good selectivity indices against cancer cells would have provided better insights into the therapeutic potential of *T. tetraptera*. However, this was not investigated, which is a limitation of this study, and future studies would be targeted at investigating this.

This is important because the identification and isolation of the compounds in the fractions responsible for the antiproliferative activity and with good selectivity indices would be beneficial for pharmacological purposes.

## Figures and Tables

**Table 1:** Phytochemical constituents of crude extract and fractions of *T. tetraptera* fruit.

Phytochemicals	Crude extract	C1	C2	C3	C4	C5	C6	C7	C8	C9
Alkaloids	+	−	+	−	−	−	+	−	+	+
Carbohydrates	+	−	−	+	−	+	+	−	+	+
Carotenoids	−	−	−	−	−	−	−	−	−	−
Flavonoids	+	−	−	−	−	−	+	+	+	+
Glycosides	+	−	+	−	+	+	−	−	−	−
Phenols	+	−	−	+	−	−	+	−	−	−
Saponins	+	−	−	−	−	−	+	+	+	+
Steroids	+	−	+	−	−	−	−	−	−	−
Tannins	+	−	+	−	−	−	+	−	+	+
Terpenoids	+	−	−	+	−	+	−	−	−	−

A plus sign (+) denotes presence, whereas a minus sign (−) denotes the absence of phytochemicals.

**Table 2:** IC_50_ (*μ*g/mL ± SD) values of crude methanolic extract and chromatographic fractions of *T. tetraptera* fruit on Chang liver, Jurkat, and MCF-7 cells.

Treatment	Chang liver	Jurkat	MCF-7
Crude extract	294.37 ± 10.91	340.37 ± 6.58	380.12 ± 29.66
C1	>1000	>1000	>1000
C2	693.09 ± 3.20	222.57 ± 9.14	593.78 ± 15.43
C3	576.55 ± 10.80	115.48 ± 5.63	302.84 ± 10.62
C4	967.91 ± 39.34	184.42 ± 2.04	663.65 ± 22.45
C5	185.91 ± 4.29	51.38 ± 3.64	190.40 ± 8.58
C6	31.86 ± 0.31	35.85 ± 2.29	70.07 ± 0.75
C7	100.98 ± 4.37	70.56 ± 3.89	152.73 ± 24.14
C8	>1000	634.56 ± 60.16	933.06 ± 16.19
C9	>1000	866.64 ± 31.24	878.63 ± 15.61
Curcumin	11.39 ± 0.59	6.96 ± 1.08	12.87 ± 1.33

The values are expressed as the mean ± standard deviation. Curcumin was used as the standard compound.

**Table 3:** Percentage content of crude and fractions from liquid chromatograph-mass spectrometry.

Crude/fraction	RT/min	% content	Some fragment ions
C1	1.839	100	101.0, 146.1, 179.1, 241.1, 319.1, 373.1
C2	1.846	100	101.1, 146.1, 179.1, 202.3, 241.1, 319.1, 373.1
C3	1.846	100	101.1, 146.1, 179.1, 241.1, 319.1, 373.1
C5	1.846	100	101.1, 146.1, 179.1, 202.3, 241.1, 319.1, 373.1
C6	1.839 10.145	87.3 12.7	101.1, 146.1, 179.1, 241.1, 319.1, 373.1 111.1, 161.2, 210.2, 261.2, 349.2, 397.3, 535.1
C7	1.372 1.595 1.839 10.145	10.28 6.35 79.68 9.69	111.1, 161.2, 223.1, 245.2, 263.2, 349.2, 393.2 89.1, 111.1, 161.2, 203.1, 245.2, 290.2, 349.2, 383.2, 435.1 101.0, 146.1, 179.1, 241.1, 319.1, 373.0 111.1, 161.2, 210.2, 261.2, 349.2, 397.3, 535.0
C8	1.609 1.861	24.38 74.62	111.1, 161.1, 203.1, 290.2, 349.2, 383.2, 445.2 101.0, 146.1, 179.0, 241.1, 319.1, 373.0, 533.4
Crude extract	1.853	100	101.1, 111.1, 161.1, 163.1, 205.1, 223.1, 261.1, 279.2 349.2, 535.0

RT: retention time.

## Data Availability

All the data supporting the results of this study are available from the corresponding author upon request.
